# Development and multicenter external validation of a deep learning model for early screening of thoracic ossification of the ligamentum flavum on routine chest radiographs

**DOI:** 10.3389/fradi.2026.1867740

**Published:** 2026-07-29

**Authors:** Zichuan Wu, Juehan Wang, Xuhong Zhang, Baifeng Sun, Min Qi, Yang Liu

**Affiliations:** 1 Department of Orthopedics, Ningbo No.6 Hospital, Ningbo, Zhejiang, China; 2 Ningbo Clinical Research Center for Orthopedics, Sports Medicine & Rehabilitation, Ningbo, Zhejiang, China; 3 Department of Orthopaedic and Reconstructive Surgery/Pediatric Orthopaedics, South China Hospital Affiliated with Shenzhen University, Shenzhen, China; 4 Department of Spine Surgery, Changzheng Hospital Affiliated to the Naval Medical University, Shanghai, China

**Keywords:** chest radiograph, deep learning, external validation, multicenter study, ResNet101, thoracic ossification of the ligamentum flavum

## Abstract

**Background:**

Thoracic ossification of the ligamentum flavum (TOLF) is frequently underrecognized in its early stage because radiographic abnormalities on routine chest radiographs are often subtle. We aimed to develop and externally validate a deep learning model for opportunistic screening of TOLF using routine chest radiographs.

**Methods:**

This retrospective multicenter diagnostic study included an internal development cohort from Changzheng Hospital and an independent external validation cohort from South China Hospital. The internal cohort comprised 250 patients with TOLF and 250 control subjects collected between January 2017 and January 2023. The external cohort comprised 150 patients with TOLF and 150 control subjects. TOLF status was established on CT using predefined radiological criteria, whereas frontal and lateral chest radiographs were used only as model inputs. We evaluated multiple backbone architectures, including ResNet101, DenseNet169, Vision Transformer, and Swin Transformer, and additionally explored three dual-view fusion strategies. Model development was performed using 10-fold cross-validation in the internal cohort, and performance was summarized using bootstrap-derived 95% confidence intervals. Human-reader comparison was conducted in the internal cohort.

**Results:**

In backbone screening within the internal cohort, ResNet101 emerged as the best-performing architecture. After subsequent input-resolution optimization, the final lateral-view ResNet101 model achieved an accuracy of 97.0%, sensitivity of 94.0%, specificity of 100.0%, and an AUC of 0.970. None of the evaluated dual-view fusion strategies outperformed the best single lateral-view model, and the poorer performance of posterior-fusion models was mainly attributable to reduced sensitivity. Compared with experienced spine surgeons and imaging physicians, the internal ResNet101 model showed significantly higher sensitivity and overall accuracy (both *p* < 0.001). In the external validation cohort, the locked model maintained robust discrimination, with an AUC of 0.954 for frontal radiographs and 0.995 for lateral radiographs. The corresponding accuracy/sensitivity/specificity values were 90.0%/84.7%/95.3% for frontal radiographs and 93.7%/89.3%/98.0% for lateral radiographs.

**Conclusion:**

A deep learning model based on routine chest radiographs may provide accurate and generalizable screening for TOLF across institutions. The lateral-view model showed the most consistent diagnostic performance, supporting its potential role as an opportunistic screening tool to prompt confirmatory CT evaluation.

## Introduction

Thoracic ossification of the ligamentum flavum (TOLF) is a potentially disabling degenerative spinal disorder that can cause progressive neurological impairment. Characterized by ectopic ossification within the ligamentum flavum, TOLF leads to a progressive narrowing of the spinal canal, potentially resulting in neurological deficits ranging from sensory disturbances to severe myelopathy and paralysis. TOLF is more prevalent in East Asian populations, suggesting a potential ethno-genetic susceptibility that requires further research (1–3). Early detection of TOLF remains challenging due to its subtle presentation and often asymptomatic onset, despite its significant clinical implications. This diagnostic ambiguity underscores the need for innovative and accessible diagnostic tools to enable timely intervention and mitigate the risk of irreversible neurological damage (4–7).

Management paradigms for TOLF are predicated on the severity of the clinical presentation and the extent of neurological compromise. While conservative measures, including pharmacologic therapy and physical rehabilitation, may suffice for mild symptomatology, advanced cases necessitating surgical intervention underscore the critical importance of timely and accurate diagnostic acumen (8).

Historically, the diagnostic workup for TOLF has relied on advanced imaging modalities, primarily computed tomography (CT) and magnetic resonance imaging (MRI), both of which provide detailed insights into the extent of spinal canal stenosis and ossification patterns. Previous CT-based epidemiological studies have shown that thoracic ligamentum flavum ossification is not uncommon in selected imaging populations; however, the proportion of clinically suspected or radiograph-detectable TOLF cases encountered during routine chest-radiograph screening is expected to be substantially lower than that in prevalence-enriched diagnostic datasets (9). Nevertheless, routine use of CT and MRI in screening asymptomatic or mildly symptomatic patients is limited by cost, accessibility, and the relatively high radiation exposure associated with CT. Consequently, many patients with early-stage TOLF remain undiagnosed until the disease progresses to a more severe stage, often following traumatic spinal cord injury (SCI). Conventional chest radiography, though commonly employed in routine physical examinations, has been underutilized in TOLF diagnosis due to its limited sensitivity in detecting subtle ossific changes. This gap highlights the potential for developing innovative diagnostic approaches that leverage widely available imaging modalities to enhance early TOLF detection.

In recent years, artificial intelligence (AI), particularly deep learning, has shown transformative potential in medical imaging. Convolutional neural networks (CNNs) and other deep learning models have achieved significant advancements in image recognition and classification, revolutionizing diagnostic radiology by enabling precise detection of complex pathological features that might elude human observers. AI-driven models, such as CheXNet for pneumonia detection and U-Net for biomedical image segmentation, exemplify how deep learning can enhance diagnostic accuracy and efficiency across various medical fields (10–13). Despite these advancements, applications of AI in spinal diagnostics remain underexplored, particularly in the context of early TOLF detection using chest radiographs. Given the high stakes of delayed diagnosis in TOLF and the limitations of current screening methods, leveraging AI's capabilities in early detection offers a promising avenue for clinical practice.

Recent advances in convolutional neural networks (CNNs) and other AI architectures have led to significant improvements in medical image analysis, facilitating the detection and classification of spinal pathologies. CNNs, such as ResNet, DenseNet, and InceptionNet, have shown superior performance in identifying spinal anomalies due to their ability to capture complex spatial hierarchies within medical image (12). In spinal radiology, early studies demonstrated the feasibility of using CNNs to detect vertebral fractures in spinal radiographs, achieving a sensitivity comparable to that of trained radiologists (14). This study underscored the capacity of CNN-based models to serve as adjuncts in radiological practice, augmenting the diagnostic capabilities of clinicians.

Building on these foundational efforts, researchers have explored the application of deep learning in diagnosing various spinal disorders, including scoliosis, degenerative disc disease, and spinal stenosis. For example, Zhang et al. developed a deep learning model for scoliosis detection from smartphone photographs, achieving high accuracy and specificity, which highlighted the versatility of AI models in non-traditional imaging sources (13). Similarly, Meng et al. introduced a model for spine alignment analysis using radiograph-comparable synthetic images, showcasing AI's adaptability across different imaging modalities to monitor and assess spinal deformities in adolescents (10).

Given the diagnostic challenges associated with TOLF, especially in its early stages, there has been increasing interest in leveraging AI for ossification detection. Ogawa et al. conducted a pilot study applying deep CNNs to plain radiographs for the detection of ossification of the posterior longitudinal ligament (OPLL) in the cervical spine, achieving promising results that suggest a similar approach may be applicable to thoracic ossification of the ligamentum flavum (11). The study demonstrated that CNNs could detect subtle ossifications that clinicians may overlook, validating the role of AI in enhancing diagnostic accuracy for early-stage ossifications.

Beyond CNNs, transformer-based architectures, such as Vision Transformer (ViT) and Swin Transformer, have been gaining attention in medical imaging due to their ability to capture long-range dependencies in images, a crucial feature for identifying distributed or subtle pathologies in complex anatomical structures like the spine. Recent studies by Dosovitskiy et al. and Liu et al. highlighted the strong performance of transformer-based models in medical image segmentation and classification tasks (15, 16). Their adaptability to high-resolution images has made them promising tools for spine imaging, as they allow for the precise delineation of anatomical features essential for the diagnosis of conditions such as TOLF.

Despite these advances, the use of AI in spinal diagnostics is still evolving, with challenges remaining in areas such as dataset heterogeneity, model interpretability, and integration into clinical workflows. Visual interpretability is particularly relevant in spinal pathology, as clinicians require not only diagnostic predictions but also insights into the areas of interest highlighted by the model. The advent of techniques such as Gradient-weighted Class Activation Mapping (Grad-CAM) has allowed researchers to visualize model attention areas, providing interpretability to CNNs and increasing clinician trust in AI-assisted diagnostics (17).

Building on these considerations, the present study was designed as a retrospective multicenter diagnostic study. We developed the model in an internal cohort collected at Changzheng Hospital and subsequently tested the locked model in an independent external cohort from South China Hospital to assess cross-institutional generalizability.

Specifically, we evaluated convolutional neural network- and transformer-based architectures on frontal and lateral chest radiographs, explored whether dual-view fusion could provide incremental value over single-view analysis, and compared the best-performing internal model with experienced human readers. We hypothesized that chest-radiograph-based deep learning could enable opportunistic screening for TOLF in routine clinical practice, while independent external validation would provide a more rigorous estimate of real-world robustness.

## Materials and methods

### Study design and participating centers

**Figure 1:**
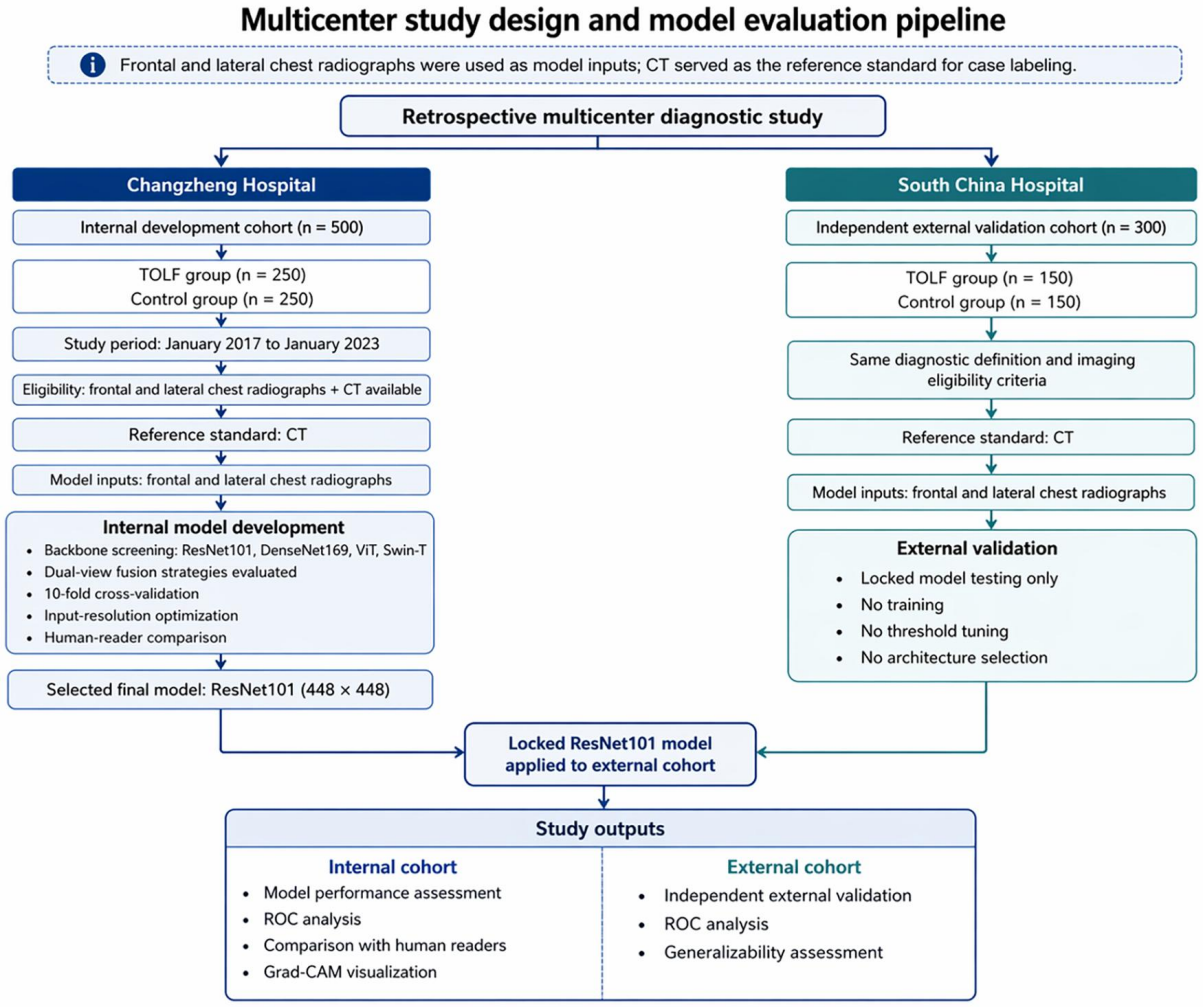
Flowchart of the multicenter study design and model evaluation pipeline. The internal development cohort from Changzheng Hospital included 250 patients with thoracic ossification of the ligamentum flavum (TOLF) and 250 control subjects and was used for model development, architecture selection, internal cross-validation, and comparison with human readers. The independent external validation cohort from South China Hospital included 150 patients with TOLF and 150 control subjects and was reserved exclusively for locked-model testing. Frontal and lateral chest radiographs were used as model inputs, whereas CT served as the reference standard for case labeling.

This retrospective multicenter diagnostic study comprised an internal development cohort from Changzheng Hospital and an independent external validation cohort from South China Hospital. The internal cohort was used for model development, architecture selection, and internal validation, whereas the external cohort was reserved exclusively for locked-model testing. The overall multicenter study design and model evaluation pipeline are shown in Figure 1.

### Internal development cohort

At Changzheng Hospital, we retrospectively enrolled 250 patients with TOLF and 250 control subjects between January 2017 and January 2023. TOLF status was established using CT as the reference standard. Control subjects were required to have no evidence of thoracic ossification of the ligamentum flavum on CT. Frontal and lateral chest radiographs were used as model inputs rather than diagnostic reference images. All labels were reviewed by an experienced spine surgeon and a musculoskeletal radiologist.

### External validation cohort

To evaluate model generalizability, we assembled an independent external cohort from South China Hospital, comprising 150 patients with TOLF and 150 control subjects. The same diagnostic definition and imaging eligibility criteria used in the internal cohort were applied to the external cohort. The external cohort was not used for model training, threshold tuning, architecture selection, or any other stage of model development.

### Reference standard and CT-based diagnostic criteria

TOLF status was determined on CT images before any radiograph-based model training or human-reader assessment. TOLF was defined as a focal or segmental hyperattenuating ossified mass arising from the ligamentum flavum along the posterior wall of the thoracic spinal canal, with continuity to the lamina-facet complex and corresponding narrowing of the posterior canal on axial CT. Sagittal and coronal reconstructions were reviewed to confirm the involved vertebral level and to distinguish ligamentum-flavum ossification from facet osteophytes, laminar sclerosis, vascular or soft-tissue calcification, and non-ossified ligamentum flavum hypertrophy. The lower thoracic and thoracolumbar junctional levels were specifically reviewed because TOLF frequently involves the lower thoracic spine. Each CT label was assigned independently by an experienced spine surgeon and a musculoskeletal radiologist, and disagreements were resolved by consensus. CT morphology was documented descriptively according to the Sato classification, including lateral, extended, enlarged, fused, and tuberous types, but subtype information was not used as a model input or training target (18, 19).

### Inclusion and exclusion criteria

Eligible subjects had available frontal and lateral chest radiographs together with CT examinations suitable for reference labeling. Patients with congenital spinal deformity or a history of thoracic spine surgery were excluded. Cases lacking complete analyzable imaging data were not entered into the final dataset. Radiographs were excluded before model development if the CT-confirmed ossified level was outside the radiographic field of view, if the lower thoracic spine was truncated at the caudal edge, or if severe rotation, poor exposure, or overlying artifacts prevented reliable visualization of the thoracic spine region.

Baseline demographic and clinical variables were collected to characterize the two cohorts and to assess group comparability between TOLF-positive and control subjects.

## Image preprocessing

All chest radiographs were exported from the institutional Picture Archiving and Communication System in JPG format. The thoracic spine region of interest was cropped using a standardized, quality-controlled preprocessing procedure rather than a fully automatic black-box crop. On lateral radiographs, the cranial boundary was placed above the upper thoracic spine when visible, and the caudal boundary was required to include T12–L1 or the lowest visible thoracolumbar junction, with the posterior vertebral body margins, pedicles, rib contours, and the 12th rib used as anatomical landmarks. On frontal radiographs, the region of interest included the visible thoracic vertebral column and bilateral rib-head/pedicle regions, and the caudal edge was extended to include the T12 or thoracolumbar junctional region whenever visible. Frontal and lateral images were adjusted to a uniform aspect ratio of 1.2 to minimize irrelevant background information while preserving the thoracic anatomical region of interest. For model development, images from the internal cohort were organized into frontal, lateral, and dual-view datasets. Internal model performance was assessed using 10-fold cross-validation rather than a fixed train-test split. All radiographs from the same individual were kept within the same fold to avoid information leakage between training and validation subsets. External radiographs from South China Hospital underwent the same preprocessing workflow and were used exclusively for independent locked-model validation.

## Model construction

**Figure 2:**
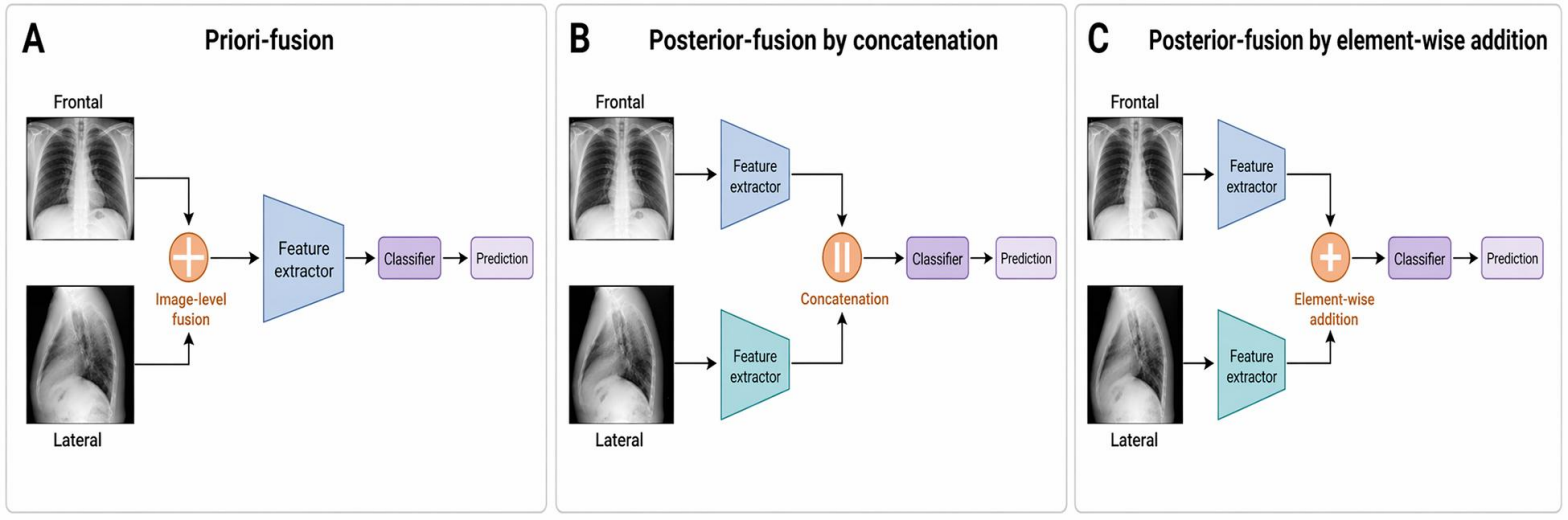
**(A)** Priori-fusion: frontal and lateral chest radiographs are combined at the image-input level and subsequently processed by a shared feature extractor before final classification. **(B)** Posterior-fusion by concatenation: frontal and lateral radiographs are processed independently by projection-specific feature extractors, and the resulting feature representations are concatenated before being passed to the classifier. **(C)** Posterior-fusion by element-wise addition: frontal and lateral radiographs are processed independently by projection-specific feature extractors, and the resulting feature representations are merged by element-wise addition before final classification. Only paired frontal and lateral radiographs from the same patient and examination were used in the dual-view fusion models.

Candidate backbone architectures included ResNet101, DenseNet169, Vision Transformer (ViT), and Swin Transformer (Swin-T). All models were initialized with ImageNet-1k pretrained weights and equipped with a binary classification head consisting of adaptive average pooling followed by a fully connected layer. To evaluate whether integrating two radiographic projections could improve performance, we additionally constructed three dual-view fusion strategies using ResNet101 as the feature extractor: priori-fusion, posterior-fusion-cat, and posterior-fusion-add. Only paired frontal and lateral radiographs from the same patient and examination were used in the fusion experiments. In the priori-fusion strategy, the two projections were resized and concatenated at the image-input level before feature extraction. In the posterior-fusion-cat and posterior-fusion-add strategies, frontal and lateral radiographs were processed by two projection-specific ResNet101 branches; the extracted feature vectors were then merged by channel concatenation or element-wise addition, respectively, before the final classifier. Decision-level fusion was not evaluated in the present study because the aim was to test whether paired image-level or feature-level integration could improve representation learning. The architectures of the evaluated dual-view fusion strategies are shown in Figure 2.

Candidate backbone architectures and dual-view fusion strategies were compared sequentially during internal model development, and detailed performance metrics are reported in the Results section. Backbone screening and input-resolution optimization were conducted as separate stages of model development. Candidate backbone architectures were first compared under a uniform baseline setting in the internal cohort for architecture selection, whereas the subsequent resolution analysis was performed only for the selected ResNet101 model. The final operating model used for ROC visualization, human-reader comparison, and external validation was the ResNet101 model at the prespecified optimal input resolution of 448 × 448.

## Training settings

Models were implemented in PyTorch and optimized using stochastic gradient descent with an initial learning rate of 0.001, momentum of 0.9, and weight decay of 0.005. A cosine annealing schedule was used for learning rate adjustment. Training was performed for 30 epochs with a batch size of 16, and cross-entropy loss was used for binary classification.

## Performance evaluation

Model performance was evaluated using the area under the receiver operating characteristic curve (AUC), accuracy, sensitivity, specificity, positive predictive value (PPV), negative predictive value (NPV), and F1 score. Candidate backbone architectures and dual-view fusion strategies were first evaluated in the internal cohort using 10-fold cross-validation. After model selection, the best-performing locked model was applied without retraining to the external validation cohort from South China Hospital. Threshold-dependent performance metrics were calculated using the prespecified operating threshold determined from the internal model-development stage and then fixed for external validation. Because both cohorts used an enriched 1:1 case-control design, we additionally performed an exploratory prevalence-scenario analysis to estimate how positive and negative predictive values would change if the locked model were applied to lower-prevalence screening populations. PPV and NPV were recalculated from the external sensitivity and specificity under hypothetical TOLF prevalences of 1%, 5%, and 10% using standard Bayes-based diagnostic-test formulas. This analysis was exploratory and did not alter the locked operating threshold. Human-reader comparison was performed only in the internal cohort because paired reader interpretations were not available in the external dataset. Grad-CAM was used to visualize model attention in representative cases. The prespecified comparison between the best-performing internal model and human readers is summarized in Table 5.

## Statistical analysis

Continuous variables are presented as mean ± standard deviation, and categorical variables are presented as counts. Baseline characteristics were compared using the independent-samples t test or the chi-square test, as appropriate. In the internal cohort, model performance was summarized across 10 cross-validation folds, and 95% confidence intervals were estimated using bootstrap resampling. ROC curves and AUCs were calculated for the frontal and lateral models. McNemar's test was used to compare the best-performing internal ResNet101 model with human readers. For the external validation cohort, the locked model's AUC, accuracy, sensitivity, specificity, PPV, NPV, and F1 score were calculated with 95% confidence intervals. All statistical tests were two-sided, and *P* < 0.05 was considered statistically significant.

## Results

### Patient characteristics

The internal development cohort comprised 500 subjects from Changzheng Hospital, including 250 patients with TOLF and 250 control subjects. The independent external validation cohort comprised 300 subjects from South China Hospital, including 150 patients with TOLF and 150 control subjects. Baseline demographic and clinical characteristics are summarized in Table 1. No significant between-group differences were observed in age, sex, body mass index, smoking history, hypertension, or diabetes in the internal cohort, and a similar pattern was observed in the external cohort (all *P* > 0.05).

**Table 1:** Baseline characteristics of the internal development cohort and the independent external validation cohort, stratified by TOLF status.

Variable	Changzheng control (*n* = 250)	Changzheng TOLF (*n* = 250)	*P* value	South China Hospital control (*n* = 150)	South China Hospital TOLF (*n* = 150)	*P* value
Age, years	46.16 ± 11.44	45.76 ± 11.60	0.698	45.80 ± 11.73	45.34 ± 11.30	0.730
Sex, male/female	122/128	112/138	0.370	63/87	56/94	0.479
BMI, kg/m^2^	26.41 ± 4.55	26.26 ± 4.53	0.705	26.08 ± 4.58	25.71 ± 4.20	0.466
Smoking history, yes/no	182/68	195/55	0.177	108/42	116/34	0.353
Hypertension, yes/no	170/80	176/74	0.561	103/47	104/46	1.000
Diabetes, yes/no	197/53	194/56	0.745	124/26	112/38	0.121

Data are presented as mean ± standard deviation or number of patients. *P* values were calculated using the independent-samples t test for continuous variables and the chi-square test for categorical variables. The internal development cohort was derived from Changzheng Hospital, and the independent external validation cohort was derived from South China Hospital. TOLF, thoracic ossification of the ligamentum flavum; BMI, body mass index.

### Model performance, comparison with human readers, and external validation

**Figure 3:**
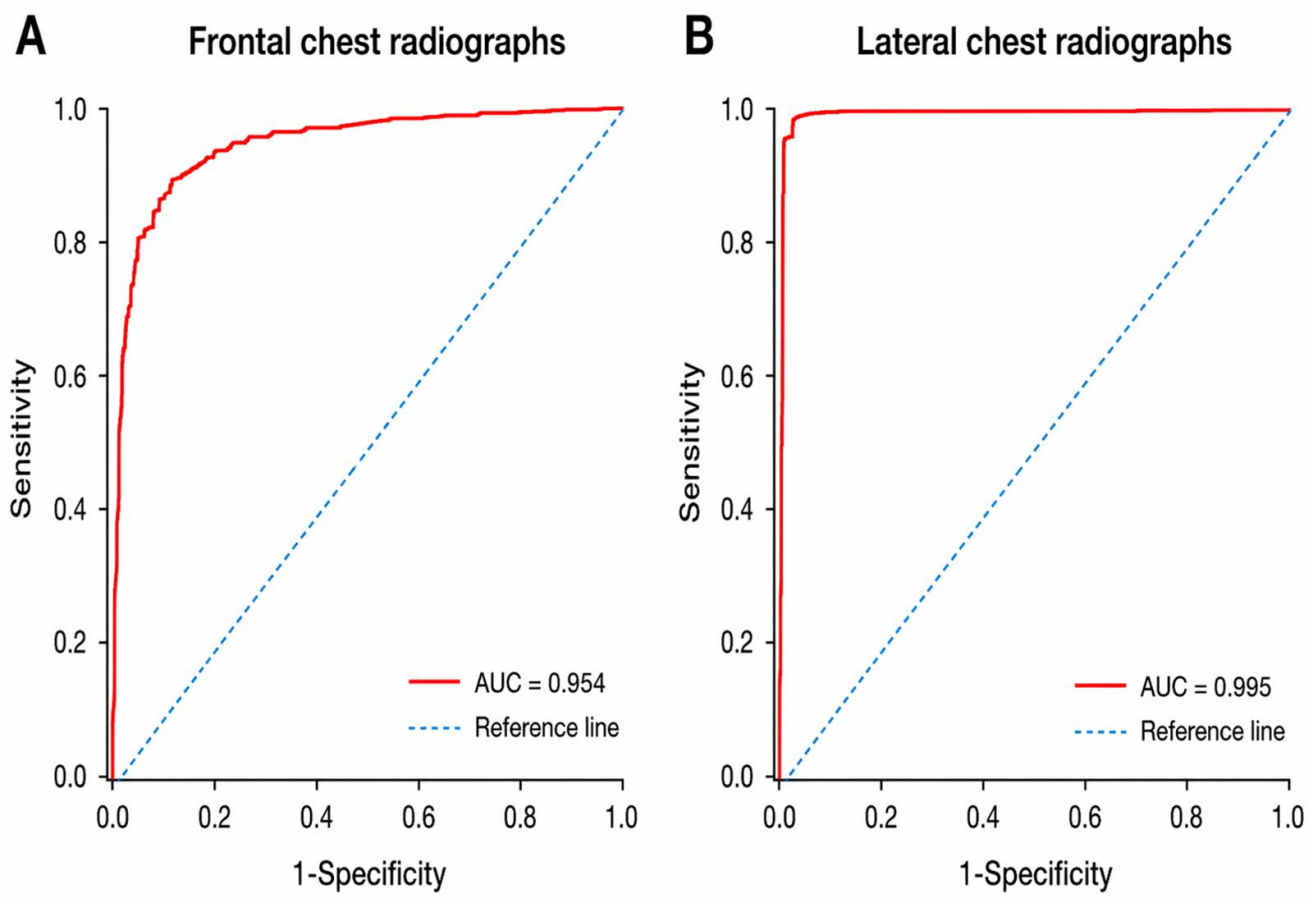
Receiver operating characteristic curves of the final internally validated ResNet101 model in the changzheng hospital cohort. **(A)** ROC curve for frontal chest radiographs. **(B)** ROC curve for lateral chest radiographs. The model was developed and internally validated in the Changzheng Hospital cohort after backbone screening and subsequent input-resolution optimization. The diagonal blue line indicates random classification performance, whereas the red curve represents the diagnostic performance of the final model.

Among the candidate backbone architectures evaluated in the internal development cohort, ResNet101 showed the best overall diagnostic performance and was therefore selected for subsequent model refinement. The comparative performance of all candidate backbone architectures under the backbone-screening setting is summarized in Table 2. After input-resolution optimization, the final ResNet101 model achieved its best performance on lateral radiographs. Receiver operating characteristic curves of the final internally validated ResNet101 model for the frontal and lateral datasets are shown in Figure 3. For the final lateral-view model, the AUC was 0.970, with an accuracy of 97.0%, sensitivity of 94.0%, and specificity of 100.0%. For the final frontal-view model, the corresponding AUC was 0.930, with an accuracy of 93.0%, sensitivity of 88.0%, and specificity of 98.0%.

**Table 2:** Performance of candidate backbone architectures in the internal development cohort.

Model	Dataset	Accuracy	Sensitivity	Specificity
ResNet101	frontal	0.902	0.880	0.924
DenseNet169	frontal	0.824	0.728	0.920
ViT	frontal	0.850	0.788	0.912
Swin-T	frontal	0.862	0.824	0.900
ResNet101	lateral	0.938	0.924	0.952
DenseNet169	lateral	0.838	0.772	0.904
ViT	lateral	0.872	0.844	0.900
Swin-T	lateral	0.880	0.832	0.928

Performance was evaluated in the internal development cohort from Changzheng Hospital using 10-fold cross-validation. Data are presented as mean values across folds. Frontal and lateral indicate the corresponding chest-radiograph views used as model inputs. ResNet101, Residual Network-101; DenseNet169, densely connected convolutional network-169; ViT, Vision Transformer; Swin-T, Swin Transformer. Higher values indicate better diagnostic performance.

Three dual-view fusion strategies were also evaluated in the internal development cohort, and their comparative performance is summarized in Table 3. Priori-fusion achieved an accuracy, sensitivity, and specificity of 0.893, 0.849, and 0.940, respectively. In contrast, posterior-fusion by feature concatenation and posterior-fusion by element-wise addition showed a marked reduction in sensitivity, with values of 0.547 and 0.340, respectively, despite preserved specificity of 0.920 and 0.960. Thus, the performance decline of the posterior-fusion models was driven primarily by missed TOLF-positive cases rather than by excessive false-positive predictions. These findings indicate that simple dual-view fusion did not provide incremental discriminative information beyond the lateral projection and may have diluted the lesion-specific features learned from lateral radiographs.

**Table 3:** Performance of dual-view fusion strategies in the internal development cohort.

Model	Method	Accuracy	Sensitivity	Specificity
FusionNet1	priori-fusion	**0** **.** **893**	**0**.**849**	**0**.**940**
FusionNet2	posterior-fusion-cat	0.728	0.547	0.920
FusionNet3	posterior-fusion-add	0.641	0.340	0.960

Dual-view fusion models were evaluated in the internal development cohort from Changzheng Hospital using paired frontal and lateral chest radiographs. Priori-fusion denotes image-level fusion before feature extraction; posterior-fusion-cat denotes feature-level fusion by channel concatenation; posterior-fusion-add denotes feature-level fusion by element-wise addition. None of the evaluated fusion strategies outperformed the best single lateral-view ResNet101 model. Bold values indicate the highest value for each performance metric among the evaluated dual-view fusion strategies.

The effect of input resolution on ResNet101 performance in the internal development cohort is summarized in Table 4. Diagnostic performance improved as the input resolution increased from 224 × 224 to 448 × 448, particularly for lateral radiographs. At 448 × 448, the lateral-view model achieved the best overall balance of accuracy, sensitivity, and specificity, whereas no meaningful additional gain was observed at 672 × 672. Additional diagnostic indices of the best-performing internal model were PPV of 1.000, NPV of 0.943, and F1 score of 0.969 for lateral radiographs, and PPV of 0.978, NPV of 0.891, and F1 score of 0.926 for frontal radiographs.

**Table 4:** Effect of input resolution on ResNet101 performance in the internal development cohort.

Resolution	Dataset	Accuracy	Sensitivity	Specificity
224 × 224	frontal	0.880	0.820	0.940
448 × 448	frontal	0.930	0.880	0.980
672 × 672	frontal	0.929	0.880	*0.981*
224 × 224	lateral	*0.950*	**0** **.** **960**	0.940
448 × 448	lateral	**0**.**970**	*0.940*	**1**.**000**
672 × 672	lateral	**0**.**970**	*0.940*	**1**.**000**

Model performance at different input resolutions was evaluated in the internal development cohort from Changzheng Hospital using the ResNet101 architecture. Frontal and lateral indicate the corresponding chest-radiograph views used as model inputs. Data are presented as mean values across cross-validation folds. The 448 × 448 setting was selected as the principal operating resolution for subsequent analyses because it provided strong overall performance without further gain at higher resolution. Bold values indicate the highest value for each performance metric across all evaluated input resolutions and radiographic views; tied highest values are all shown in bold. Italic values indicate the performance metrics obtained at the selected input resolution of 448 × 448, which was used for subsequent model evaluation and validation.

The comparison between the best-performing internal ResNet101 model and human readers in the Changzheng Hospital cohort is summarized in Table 5. The internally validated ResNet101 model achieved substantially higher sensitivity and overall accuracy than both spine surgeons and imaging physicians for frontal and lateral radiographs. A ROC-based visual comparison between the internally validated ResNet101 model and the participating human readers is provided in Supplementary Figure S1. Because human readers provided binary TOLF/no-TOLF judgments rather than continuous confidence scores, full reader-specific ROC curves were not generated. Instead, each reader was plotted as a single operating point in the model ROC space according to sensitivity and 1-specificity. The operating-point data used for this ROC-based visual comparison are provided in Supplementary Table S1. McNemar's test showed significant differences between the model and human readers for both views (both *P* < 0.001).

**Table 5:** Comparison between the best-performing internal ResNet101 model and human readers in the Changzheng Hospital cohort.

Assessor	View	Accuracy	Sensitivity	Specificity
Spine surgeon 1	frontal	0.590	0.400	0.780
Spine surgeon 2	frontal	0.660	0.420	0.900
Spine surgeon 3	frontal	0.620	0.440	0.800
Imaging physician 1	frontal	0.580	0.340	0.820
Imaging physician 2	frontal	0.600	0.340	0.860
Imaging physician 3	frontal	0.610	0.340	0.880
ResNet101	frontal	0.930	0.880	0.980
Spine surgeon 1	lateral	0.730	0.500	0.960
Spine surgeon 2	lateral	0.740	0.520	0.960
Spine surgeon 3	lateral	0.760	0.580	0.940
Imaging physician 1	lateral	0.690	0.440	0.940
Imaging physician 2	lateral	0.740	0.520	0.960
Imaging physician 3	lateral	0.700	0.500	0.900
ResNet101	lateral	0.970	0.940	1.000

Human-reader comparison was performed only in the internal development cohort from Changzheng Hospital. Spine surgeons and imaging physicians independently assessed the radiographs without access to model outputs. The ResNet101 model shown here represents the best-performing internally validated model. Frontal and lateral indicate the corresponding chest-radiograph views used for assessment.

**Figure 4:**
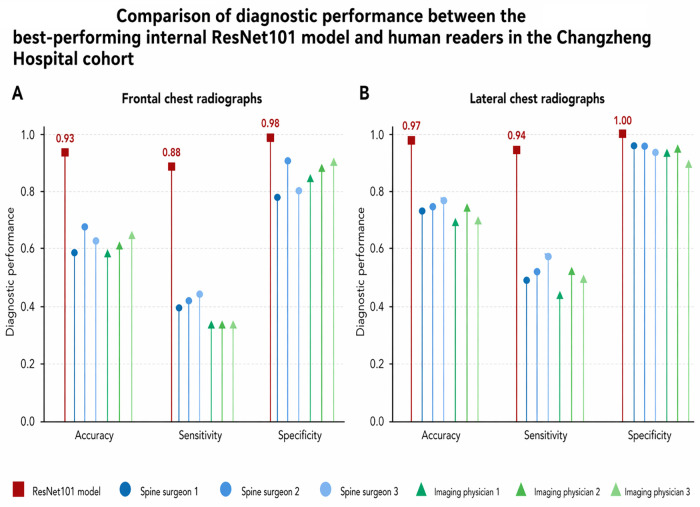
**(A)** Diagnostic performance on frontal chest radiographs. **(B)** Diagnostic performance on lateral chest radiographs. Accuracy, sensitivity, and specificity are presented for the ResNet101 model and six human readers, comprising three spine surgeons and three imaging physicians. Red squares represent the ResNet101 model, blue circles represent the spine surgeons, and green triangles represent the imaging physicians. Each human-reader symbol represents the performance of an individual reader. The numerical values above the red squares indicate the corresponding performance metrics of the ResNet101 model.

External validation in the South China Hospital cohort demonstrated preservation of model performance in an independent dataset. The external validation results are summarized in Table 6. Receiver operating characteristic curves for the locked model in the external cohort are shown in Figure 4. For frontal radiographs, the model achieved an AUC of 0.954, accuracy of 90.0%, sensitivity of 84.7%, specificity of 95.3%, PPV of 94.8%, NPV of 86.1%, and F1 score of 89.4%. For lateral radiographs, the corresponding values were 0.995, 93.7%, 89.3%, 98.0%, 97.8%, 90.2%, and 93.4%, respectively. Because the validation cohorts were prevalence-enriched, the observed PPV should not be interpreted as the expected PPV in routine chest-radiograph screening. In the prevalence-scenario analysis, the lateral-view model, using the external sensitivity of 89.3% and specificity of 98.0%, yielded projected PPVs of 31.1%, 70.1%, and 83.2% and projected NPVs of 99.9%, 99.4%, and 98.8% at assumed TOLF prevalences of 1%, 5%, and 10%, respectively. For the frontal-view model, the corresponding PPVs were 15.4%, 48.7%, and 66.7%, and the NPVs were 99.8%, 99.2%, and 98.2%, respectively (Supplementary Table S2). These estimates suggest that the lateral-view model would retain a high rule-out value in low-prevalence settings, whereas positive alerts should be interpreted as prompts for clinical review and confirmatory CT rather than as definitive diagnostic labels.

**Table 6:** External validation performance of the locked ResNet101 model in the independent South China Hospital cohort.

View	AUC (95% CI)	Accuracy (95% CI)	Sensitivity (95% CI)	Specificity (95% CI)	PPV (95% CI)	NPV (95% CI)	F1 score (95% CI)
Frontal	0.954 (0.928–0.975)	0.900 (0.863–0.933)	0.847 (0.784–0.901)	0.953 (0.917–0.981)	0.948 (0.906–0.980)	0.861 (0.808–0.910)	0.894 (0.853–0.930)
Lateral	0.995 (0.990–0.999)	0.937 (0.907–0.963)	0.893 (0.842–0.940)	0.980 (0.955–1.000)	0.978 (0.950–1.000)	0.902 (0.854–0.943)	0.934 (0.901–0.962)

The external validation cohort from South China Hospital was reserved exclusively for locked-model testing and was not used for model training, threshold tuning, architecture selection, or internal cross-validation. Performance is reported separately for frontal and lateral chest radiographs. AUC, area under the receiver operating characteristic curve; PPV, positive predictive value; NPV, negative predictive value; CI, confidence interval. Confidence intervals were estimated by bootstrap resampling.

**Figure 5:**
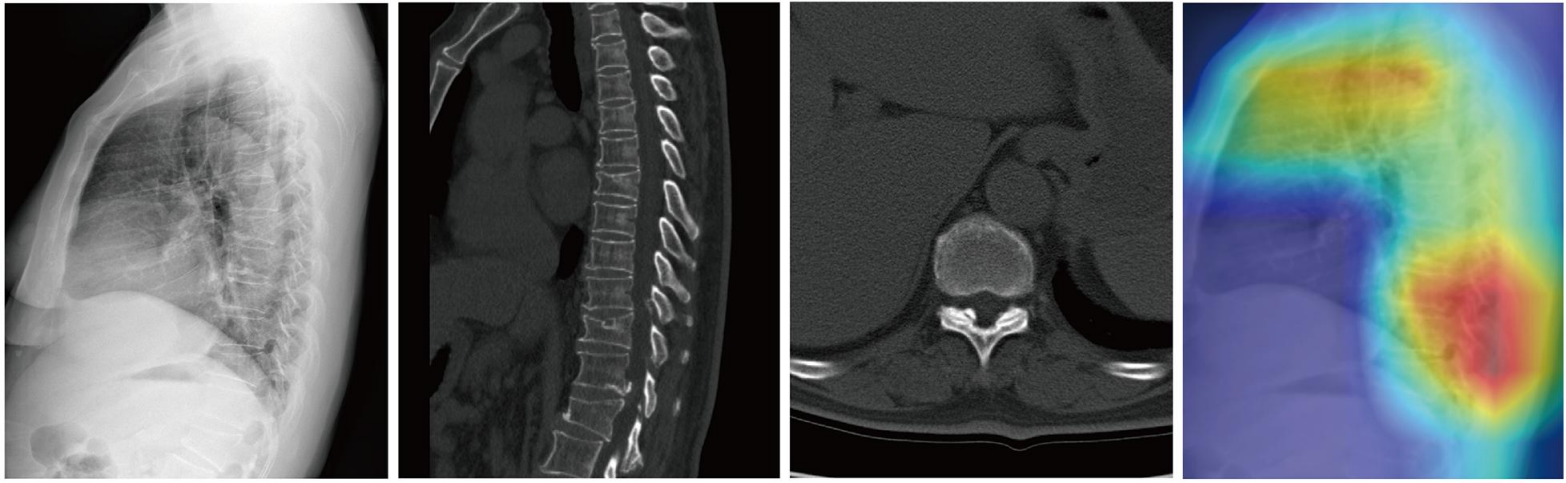
Representative case illustrating radiographic, CT, and grad-CAM findings of thoracic ossification of the ligamentum flavum. From left to right, the panels show a lateral chest radiograph, sagittal CT, axial CT, and the corresponding Grad-CAM heatmap generated by the lateral-view ResNet101 model. The highlighted region on the heatmap corresponds to the posterior thoracic canal lesion identified on CT, indicating that the model focused on the anatomically relevant area associated with TOLF.

Grad-CAM visualization of a representative case from the lateral-view model is shown in Figure 5. The heatmap localized to the lower thoracic posterior canal region corresponding to the ossified lesion identified on the sagittal and axial CT images, whereas the lesion was only subtly visible on the lateral chest radiograph. This finding supports the anatomical plausibility of the model's prediction.

## Discussion

TOLF remains clinically important because delayed recognition may allow progressive thoracic canal compromise to remain undetected until neurological dysfunction becomes substantial or acute deterioration occurs after minor trauma (3, 4, 9). In this context, the main contribution of the present study is not to redefine diagnostic standards, but to show that routinely acquired chest radiographs may contain sufficient information for AI-assisted opportunistic screening, particularly when lateral-view images are analyzed using a carefully optimized deep learning model (20–24).

The present study underscores the transformative potential of artificial intelligence (AI) in advancing the diagnostic capabilities for thoracic ossification of the ligamentum flavum (TOLF), a condition often underdiagnosed in its early stages due to the limitations of conventional radiographic methods. By evaluating deep learning models on routine frontal and lateral chest radiographs, this study introduces a practical and scalable approach to opportunistic TOLF screening using widely available imaging. A major strength of the revised study is the independent external validation using data from South China Hospital. The preservation of high discrimination in this cohort, particularly for lateral radiographs, suggests that the model captured radiographic patterns that are not confined to a single institutional workflow. This point is clinically important, because internally optimized performance in diagnostic AI studies often overestimates real-world utility. By demonstrating stable performance in an independent cohort, the present study provides stronger evidence for potential clinical generalizability.

The principal value of the present model is not to replace CT or MRI, which remain the reference imaging tools for definitive diagnosis and surgical planning, but to improve opportunistic screening on routine chest radiographs and thereby identify patients who may benefit from confirmatory cross-sectional imaging. Traditional imaging modalities, while effective, are limited by accessibility, cost, and practicality for widespread screening, especially in asymptomatic or mildly symptomatic individuals. In contrast, the proposed model can be applied to routine chest radiographs, which are ubiquitous in clinical practice and available in most healthcare facilities. By facilitating the early identification of TOLF in a cost-effective manner, this AI-based approach holds significant potential for improving patient outcomes by enabling timely intervention before the disease progresses to severe neurological impairment.

## Impact and clinical advantages

Implementing our model in a clinical setting can substantially impact diagnostic workflows by enhancing the diagnostic sensitivity of routine chest radiographs for TOLF detection. This improvement is particularly pertinent given that early-stage TOLF often presents with subtle radiographic features that may be overlooked by human observers (12). Our model's high sensitivity and specificity reduce the likelihood of false negatives and misdiagnosis, contributing to more accurate clinical decision-making. In the internal cohort, the best-performing model outperformed the participating human readers, suggesting that AI-assisted interpretation may help reduce under-recognition of subtle radiographic findings (10). This pattern is further illustrated in Supplementary Figure S1. As such, this AI-based diagnostic tool not only supports clinical accuracy but also promotes standardization in diagnostics, reducing variability across different clinical settings.

An important finding of the present study is that lateral chest radiographs carried the most informative features for TOLF screening. This finding is anatomically plausible. TOLF is located in the posterior thoracic canal and is more directly projected on lateral chest radiographs, whereas frontal chest radiographs are affected by overlapping ribs, pedicles, scapulae, mediastinal structures, and variable lower-thoracic coverage. Therefore, adding frontal radiographs does not necessarily introduce complementary lesion-specific information. The underperformance of the posterior-fusion models may also be related to methodological factors. Feature concatenation increases feature dimensionality relative to the paired sample size and may increase overfitting, whereas element-wise addition assumes a degree of feature correspondence between frontal and lateral projections that may not exist because the two views are not spatially co-registered. These factors may explain why posterior-fusion models became highly specific but insufficiently sensitive. Future work should explore projection-aware cross-view attention, calibrated decision-level ensembles, or transformer-based fusion strategies rather than simple concatenation or element-wise addition. Although both frontal and lateral projections were evaluated, none of the explored dual-view fusion strategies provided incremental benefit over the best single lateral-view model. This result suggests that simply combining two projections does not necessarily improve diagnostic performance and that more sophisticated multimodal integration strategies may be required if dual-view models are to outperform carefully optimized single-view approaches (11, 13).

## Scalability and integration into clinical workflow

From a translational perspective, the use of routine chest radiographs as model inputs may facilitate future integration into existing imaging workflows without requiring dedicated additional examinations. In principle, such a model could function as a screening aid or second reader within PACS-based environments, prompting targeted review or confirmatory CT when subtle but suspicious thoracic posterior canal abnormalities are detected. However, the actual clinical value of this approach will need to be established prospectively, particularly with respect to workflow efficiency, false-positive burden, and downstream imaging utilization. The enriched 1:1 case-control design should be considered when interpreting threshold-dependent metrics. Such a design is appropriate for model development and balanced internal comparison, but it does not reflect the substantially lower positivity rate expected in routine chest-radiograph screening. In a low-prevalence population, even a model with high specificity may generate a non-negligible number of false-positive alerts. For example, at an assumed prevalence of 1%, the external lateral-view model would be expected to detect approximately 89 of 100 TOLF-positive individuals per 10,000 screened radiographs, while generating approximately 198 false-positive alerts. Therefore, the model should be positioned as an opportunistic triage tool with a high negative predictive value rather than as a stand-alone diagnostic system. In practical deployment, the operating threshold may need to be calibrated according to local prevalence, radiology workload, and the acceptable balance between missed cases and confirmatory CT referrals.

## Future directions and limitations

Several limitations should be acknowledged. First, this was a retrospective two-center study, and broader geographic, ethnic, and technical heterogeneity should be evaluated in future prospective multicenter investigations. Second, paired human-reader interpretations were available only in the internal cohort. Therefore, the present study cannot determine whether the model's apparent advantage over physicians would be preserved across institutions, image-acquisition protocols, and reader backgrounds. External reader benchmarking and reader-AI interaction studies should be incorporated into future prospective validation before clinical deployment. Third, the present study focused on binary classification and did not address lesion burden, segmental extent, or disease severity. Fourth, variability in radiograph quality across institutions may influence model performance; future work should therefore consider normalization and domain-adaptation strategies. Finally, the real-world utility of this screening framework will depend on prospective assessment of workflow integration, referral thresholds for confirmatory CT, and downstream clinical benefit, including longitudinal outcome evaluation.

Another potential direction is the integration of AI explainability techniques, such as Gradient-weighted Class Activation Mapping (Grad-CAM), to enhance the transparency of the model's decision-making process. By visually highlighting the areas of interest within an image, Grad-CAM could provide clinicians with interpretative insights into the model's focus, thus fostering greater trust in AI-assisted diagnosis. This interpretability is particularly valuable in spinal pathology, where understanding the precise location and extent of ossification is critical for determining the most appropriate clinical management.

## Conclusion

In conclusion, we developed a deep learning model for TOLF screening on routine chest radiographs and externally validated it in an independent cohort from South China Hospital. The lateral-view ResNet101 model showed the most consistent diagnostic performance across institutions, whereas the current dual-view fusion strategies did not outperform the best single-view model. These findings support the potential role of AI-assisted chest-radiograph analysis as an opportunistic screening tool to identify patients who may benefit from confirmatory CT evaluation.

## Data Availability

The original contributions presented in the study are included in the article/Supplementary Material, further inquiries can be directed to the corresponding authors.

## Supplementary material

Supplementary Figure S1 ROC-based visual comparison between the best-performing internal ResNet101 model and human readers in the Changzheng Hospital cohort. **(A)** Frontal chest radiographs. **(B)** Lateral chest radiographs. The red curve represents the ResNet101 model, whereas individual human readers are shown as operating points based on their sensitivity and 1-specificity. Human readers included three spine surgeons and three imaging physicians. Because readers provided binary diagnostic judgments rather than continuous confidence scores, reader-specific ROC curves were not generated.

Supplementary Table S1 Operating-point data of the internally validated ResNet101 model and human readers in the Changzheng Hospital cohort. Data are shown as threshold-dependent operating-point metrics. For human readers, 1-specificity and sensitivity were used to plot the reader operating points in Supplementary Figure S1. Human readers provided binary TOLF/no-TOLF judgments rather than continuous confidence scores; therefore, reader-specific ROC curves and AUCs were not calculated. The ResNet101 rows indicate the operating-threshold performance of the internally validated model.

Supplementary Table S2 Projected predictive values and screening yield under different assumed TOLF prevalences. PPV and NPV were recalculated from the external validation sensitivity and specificity of the locked ResNet101 model under hypothetical TOLF prevalences of 1%, 5%, and 10%. Projected true-positive and false-positive counts were estimated per 10,000 screened radiographs. These calculations were exploratory and were intended to illustrate the potential impact of disease prevalence on real-world screening performance; they did not alter the prespecified operating threshold. TOLF, thoracic ossification of the ligamentum flavum; PPV, positive predictive value; NPV, negative predictive value.
