# Supplementary material for: Development and multicenter external validation of a deep learning model for early screening of thoracic ossification of the ligamentum flavum on routine chest radiographs
Source: Front Radiol. 2026 Jul 29;6:1867740. doi: 10.3389/fradi.2026.1867740 (PMC13461901; doi:10.3389/fradi.2026.1867740)
Supplement: Supplementary file 1 [file Table1.docx]

**Supplementary Table S1. Operating-point data of the internally validated ResNet101 model and human readers in the Changzheng Hospital cohort**

| **View** | **Assessor** | **Accuracy** | **Sensitivity** | **Specificity** | **1-Specificity** |
| --- | --- | --- | --- | --- | --- |
| Frontal | ResNet101 model | 0.930 | 0.880 | 0.980 | 0.020 |
| Frontal | Spine surgeon 1 | 0.590 | 0.400 | 0.780 | 0.220 |
| Frontal | Spine surgeon 2 | 0.660 | 0.420 | 0.900 | 0.100 |
| Frontal | Spine surgeon 3 | 0.620 | 0.440 | 0.800 | 0.200 |
| Frontal | Imaging physician 1 | 0.580 | 0.340 | 0.820 | 0.180 |
| Frontal | Imaging physician 2 | 0.600 | 0.340 | 0.860 | 0.140 |
| Frontal | Imaging physician 3 | 0.610 | 0.340 | 0.880 | 0.120 |
| Lateral | ResNet101 model | 0.970 | 0.940 | 1.000 | 0.000 |
| Lateral | Spine surgeon 1 | 0.730 | 0.500 | 0.960 | 0.040 |
| Lateral | Spine surgeon 2 | 0.740 | 0.520 | 0.960 | 0.040 |
| Lateral | Spine surgeon 3 | 0.760 | 0.580 | 0.940 | 0.060 |
| Lateral | Imaging physician 1 | 0.690 | 0.440 | 0.940 | 0.060 |
| Lateral | Imaging physician 2 | 0.740 | 0.520 | 0.960 | 0.040 |
| Lateral | Imaging physician 3 | 0.700 | 0.500 | 0.900 | 0.100 |

Data are shown as threshold-dependent operating-point metrics. For human readers, 1-specificity and sensitivity were used to plot the reader operating points in Supplementary Figure S1. Human readers provided binary TOLF/no-TOLF judgments rather than continuous confidence scores; therefore, reader-specific ROC curves and AUCs were not calculated. The ResNet101 rows indicate the operating-threshold performance of the internally validated model.

**Supplementary Table S2. Projected predictive values and screening yield under different assumed TOLF prevalences**

| **View** | **Sensitivity** | **Specificity** | **Assumed TOLF prevalence** | **Projected PPV** | **Projected NPV** | **Projected true positives per 10,000 screened radiographs** | **Projected false positives per 10,000 screened radiographs** |
| --- | --- | --- | --- | --- | --- | --- | --- |
| Frontal | 0.847 | 0.953 | 1% | 15.4% | 99.8% | 84.7 | 465.3 |
| Frontal | 0.847 | 0.953 | 5% | 48.7% | 99.2% | 423.5 | 446.5 |
| Frontal | 0.847 | 0.953 | 10% | 66.7% | 98.2% | 847.0 | 423.0 |
| Lateral | 0.893 | 0.980 | 1% | 31.1% | 99.9% | 89.3 | 198.0 |
| Lateral | 0.893 | 0.980 | 5% | 70.1% | 99.4% | 446.5 | 190.0 |
| Lateral | 0.893 | 0.980 | 10% | 83.2% | 98.8% | 893.0 | 180.0 |

PPV and NPV were recalculated from the external validation sensitivity and specificity of the locked ResNet101 model under hypothetical TOLF prevalences of 1%, 5%, and 10%. Projected true-positive and false-positive counts were estimated per 10,000 screened radiographs. These calculations were exploratory and were intended to illustrate the potential impact of disease prevalence on real-world screening performance; they did not alter the prespecified operating threshold. TOLF, thoracic ossification of the ligamentum flavum; PPV, positive predictive value; NPV, negative predictive value.
