# Supplementary material for: Development and multicenter external validation of a deep learning model for early screening of thoracic ossification of the ligamentum flavum on routine chest radiographs
Source: Front Radiol. 2026 Jul 29;6:1867740. doi: 10.3389/fradi.2026.1867740 (PMC13461901; doi:10.3389/fradi.2026.1867740)
Supplement: Supplementary file 2 [file Table2.docx]

**Supplementary Table S2. Projected predictive values and screening yield under different assumed TOLF prevalences**

| **View** | **Sensitivity** | **Specificity** | **Assumed TOLF prevalence** | **Projected PPV** | **Projected NPV** | **Projected true positives per 10,000 screened radiographs** | **Projected false positives per 10,000 screened radiographs** |
| --- | --- | --- | --- | --- | --- | --- | --- |
| Frontal | 0.847 | 0.953 | 1% | 15.4% | 99.8% | 84.7 | 465.3 |
| Frontal | 0.847 | 0.953 | 5% | 48.7% | 99.2% | 423.5 | 446.5 |
| Frontal | 0.847 | 0.953 | 10% | 66.7% | 98.2% | 847.0 | 423.0 |
| Lateral | 0.893 | 0.980 | 1% | 31.1% | 99.9% | 89.3 | 198.0 |
| Lateral | 0.893 | 0.980 | 5% | 70.1% | 99.4% | 446.5 | 190.0 |
| Lateral | 0.893 | 0.980 | 10% | 83.2% | 98.8% | 893.0 | 180.0 |

PPV and NPV were recalculated from the external validation sensitivity and specificity of the locked ResNet101 model under hypothetical TOLF prevalences of 1%, 5%, and 10%. Projected true-positive and false-positive counts were estimated per 10,000 screened radiographs. These calculations were exploratory and were intended to illustrate the potential impact of disease prevalence on real-world screening performance; they did not alter the prespecified operating threshold. TOLF, thoracic ossification of the ligamentum flavum; PPV, positive predictive value; NPV, negative predictive value.
